# Supplementary figures and images for: Multimodal Ageing Biomarkers and Plasma Proteomic Signatures Associated with All-Cause Mortality
Source: medRxiv. 2026 Mar 10:2026.03.09.26347914. Preprint. [Version 1] doi: 10.64898/2026.03.09.26347914 (PMC13019151; doi:10.64898/2026.03.09.26347914)

A

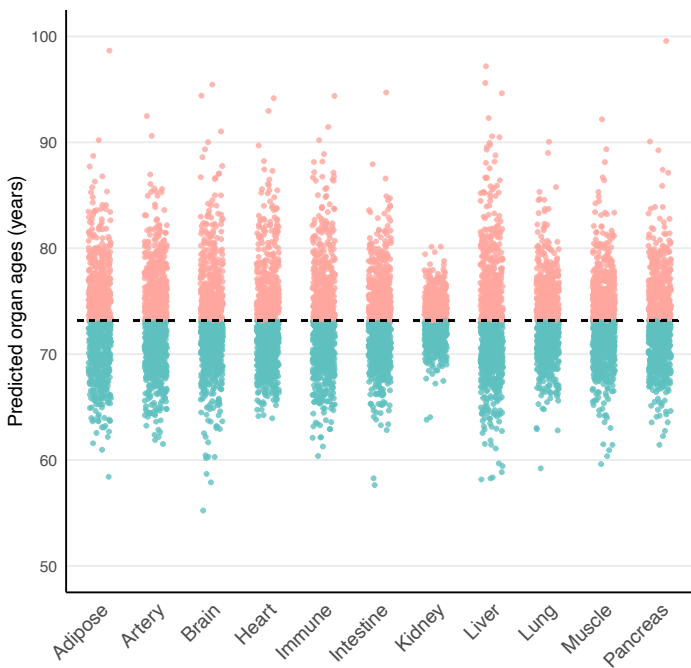

B

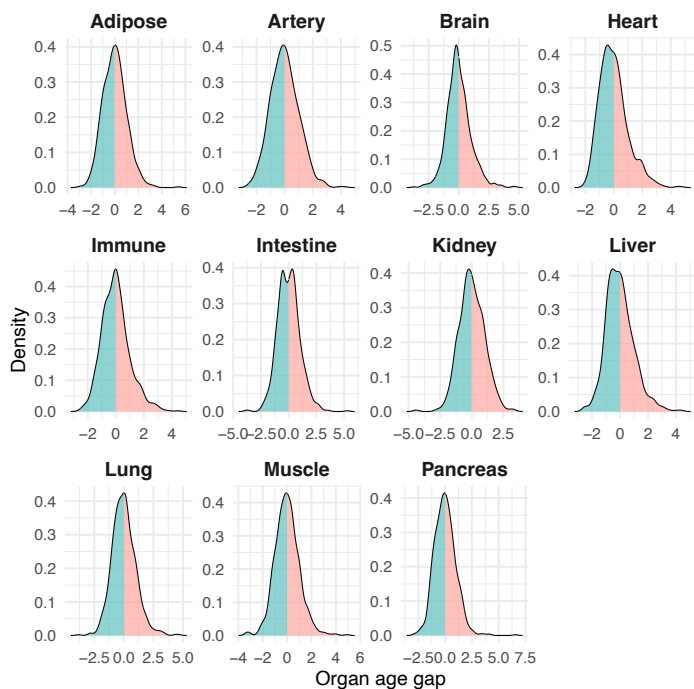

C

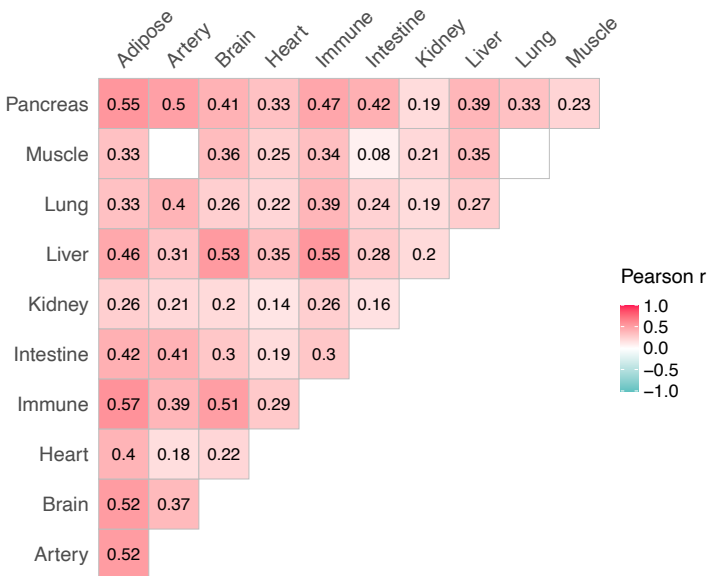

D

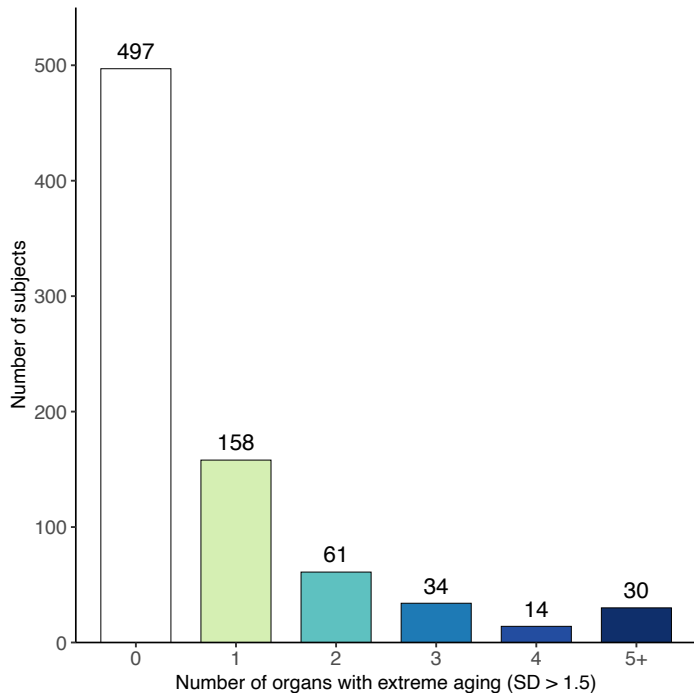

Supplement: Supplement 1 — (A) Predicted ages for eleven organs in LBC1936 wave 2 participants (N = 795). Each point represents an individual, and the black dashed line indicates the mean organ age. (B) Density plots of organ age gaps, defined as the standardised residuals (mean = 0, SD = 1) from the linear regression of predicted organ age on chronological age. Positive values, indicating accelerated ageing, are shown in pink, and negative values, indicating slower ageing, are shown in green. (C) Pairwise Pearson correlation matrix of organ age gaps; only significant correlations with PFDR < 0.05 are displayed. Positive correlations are shown in pink, and negative correlations in green. (D) Number of individuals exhibiting extreme organ ageing (standard deviation > 1.5) in a single or multiple organs. [file media-1.pdf]

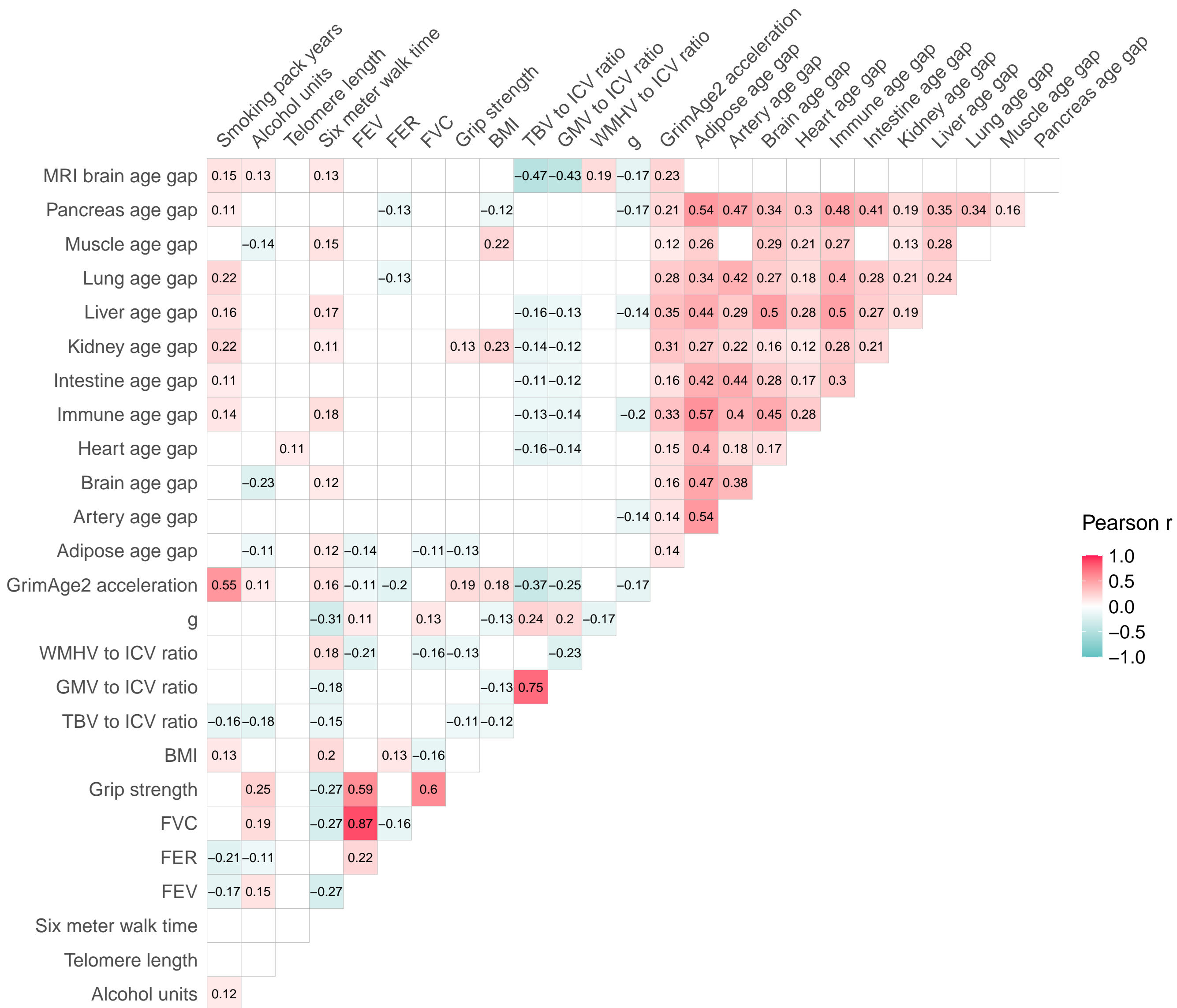

Supplement: Supplement 2 — Most biomarkers are weakly (|r| ≤ 0.4) to moderately (0.4 < |r| ≤ 0.7) correlated, with strong correlations (|r| > 0.7) observed only between forced expiratory ratio (FER) and forced expiratory volume (FEV), and between grey matter volume (GMV) and total brain volume (TBV). Red squares indicate positive correlations, and green squares indicate negative correlations. Only significant associations are displayed (PFDR < 0.05). [file media-2.pdf]

GDF15

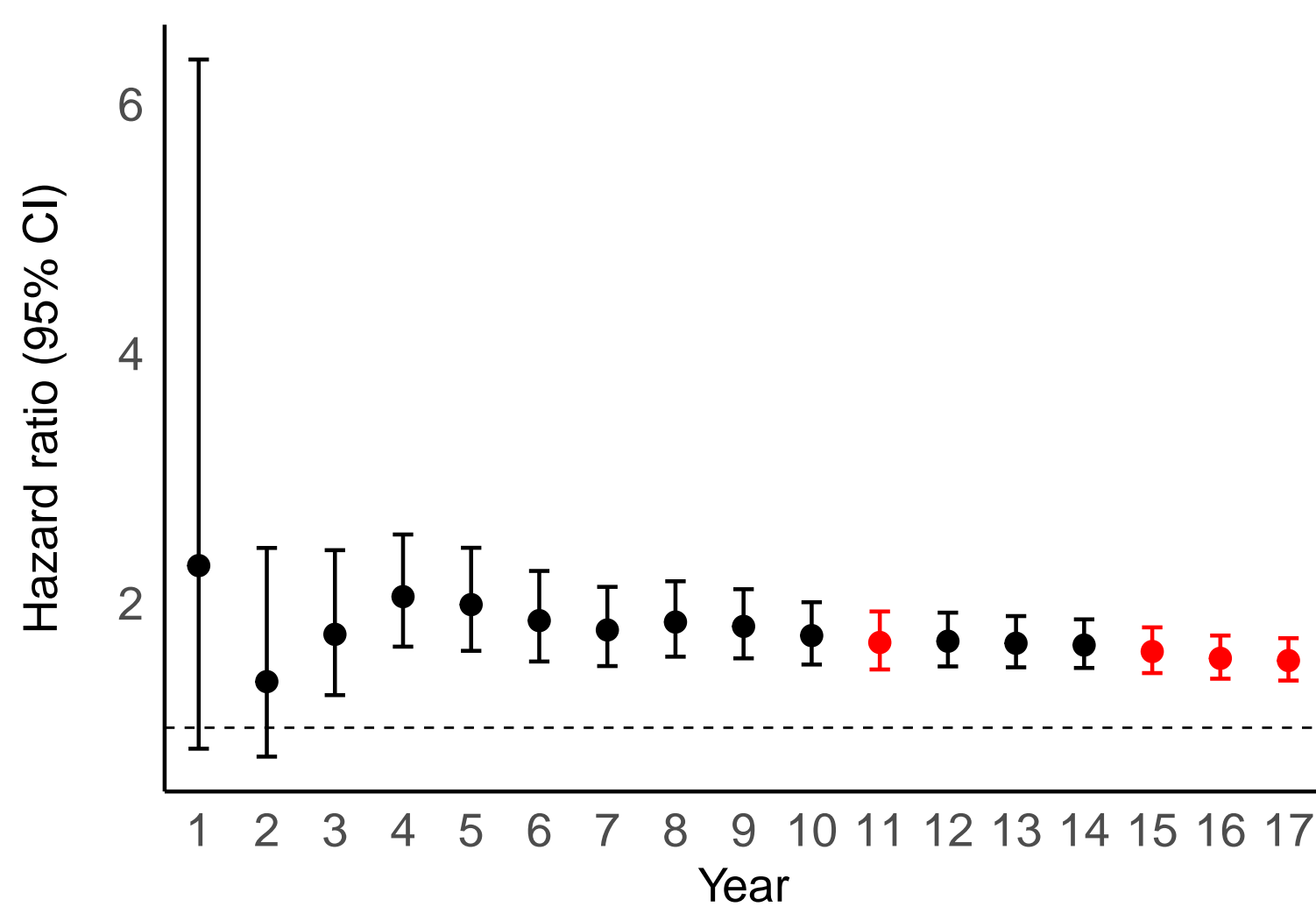

CST3

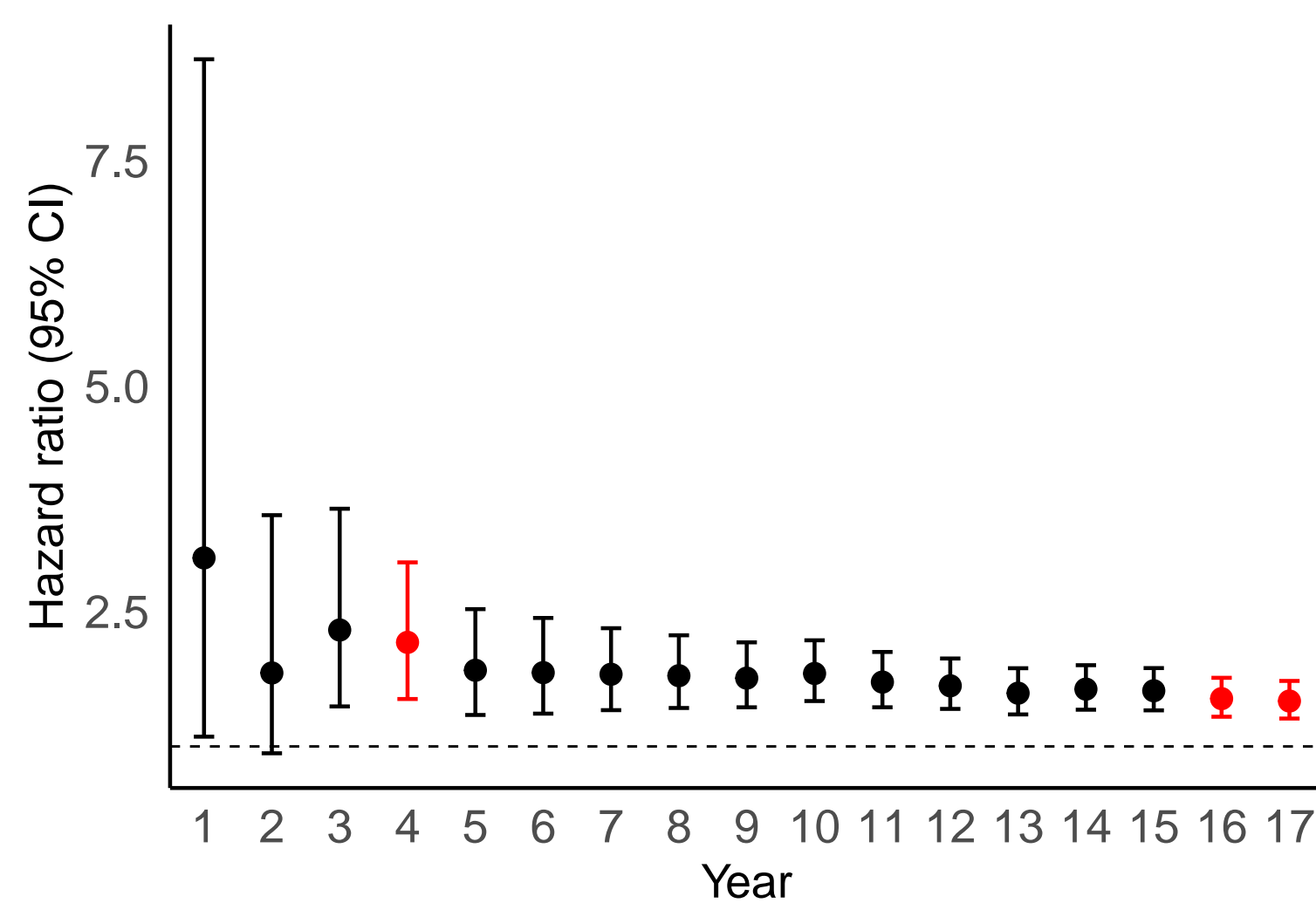

WFDC2

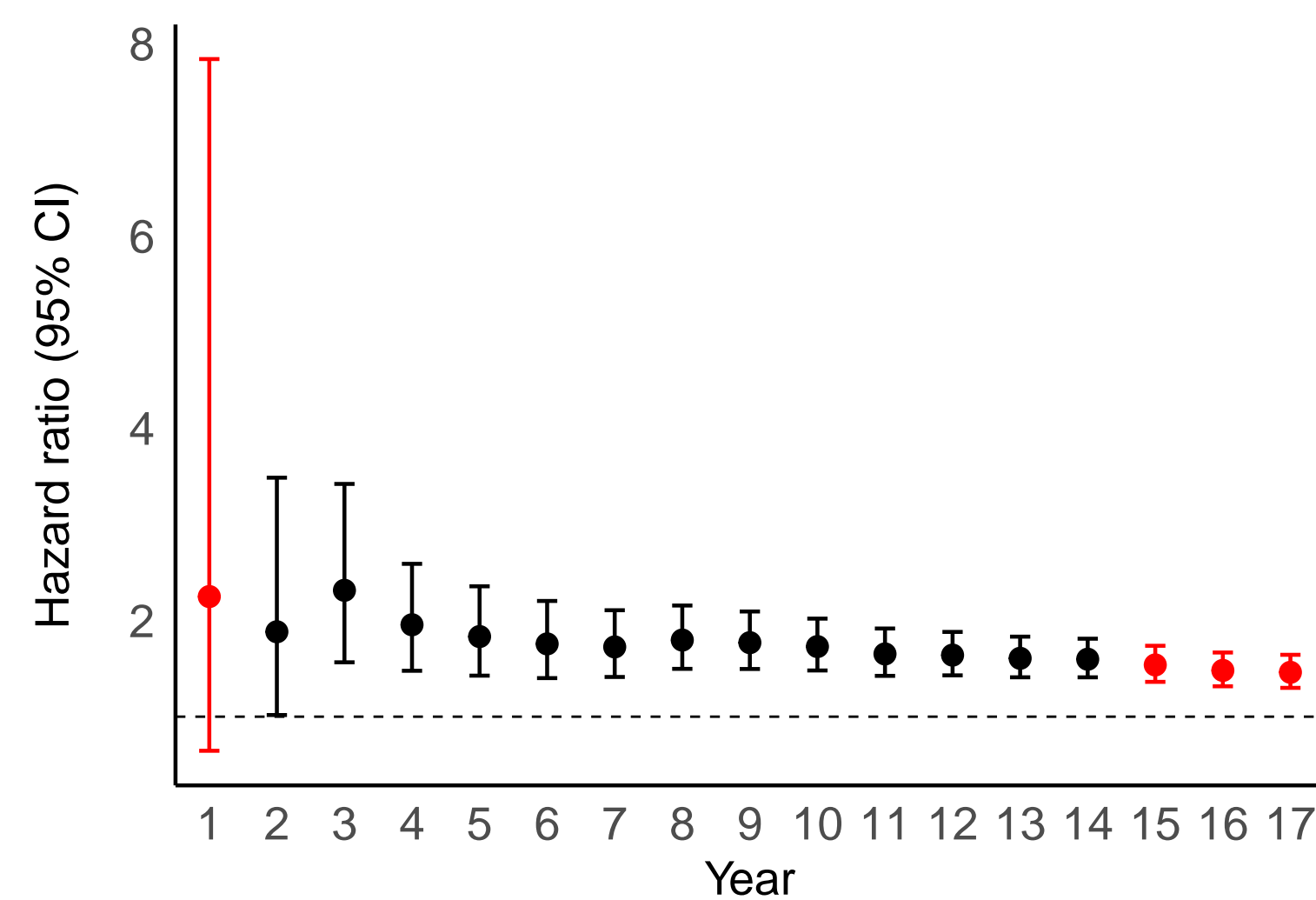

NOX4

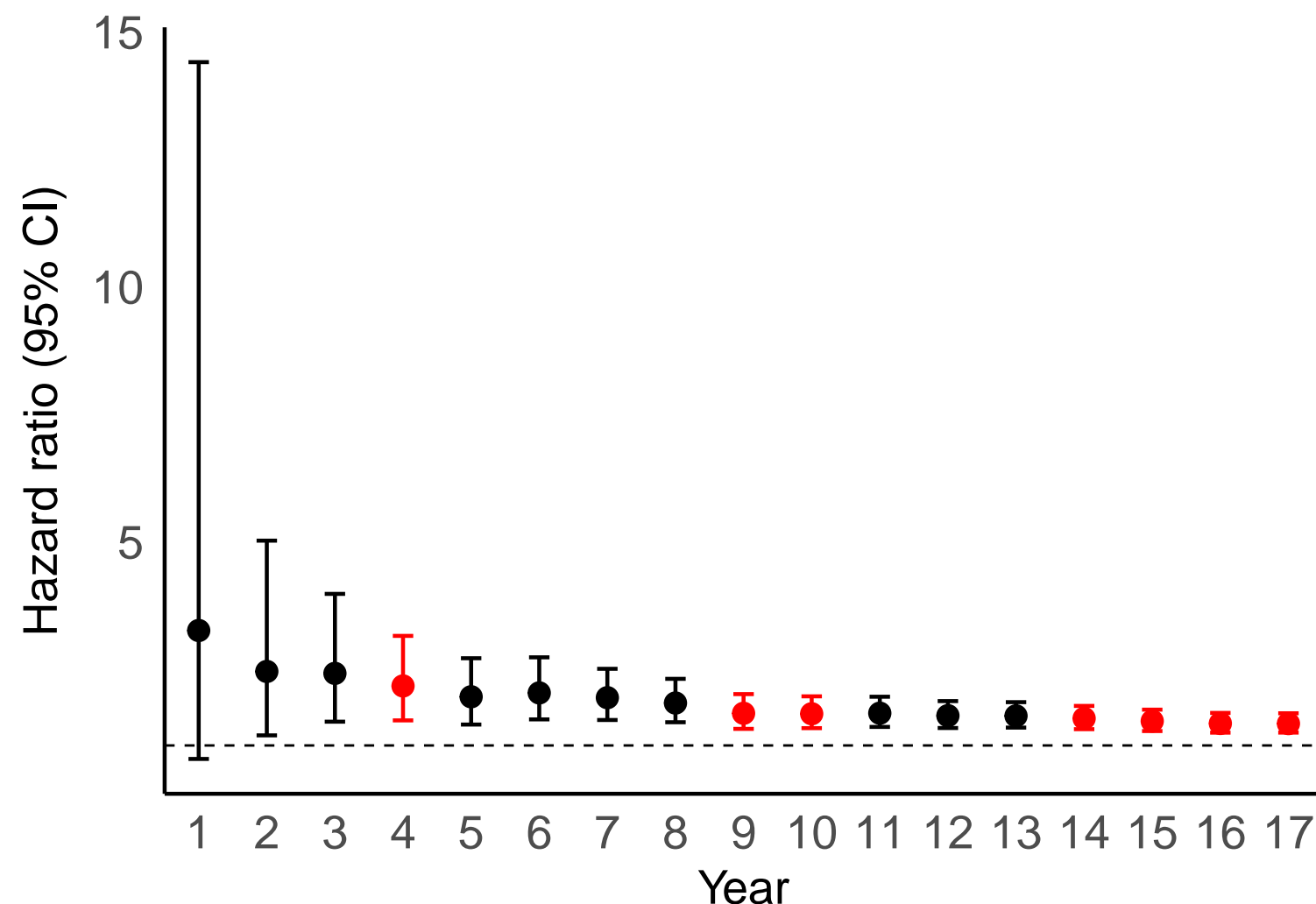

C7

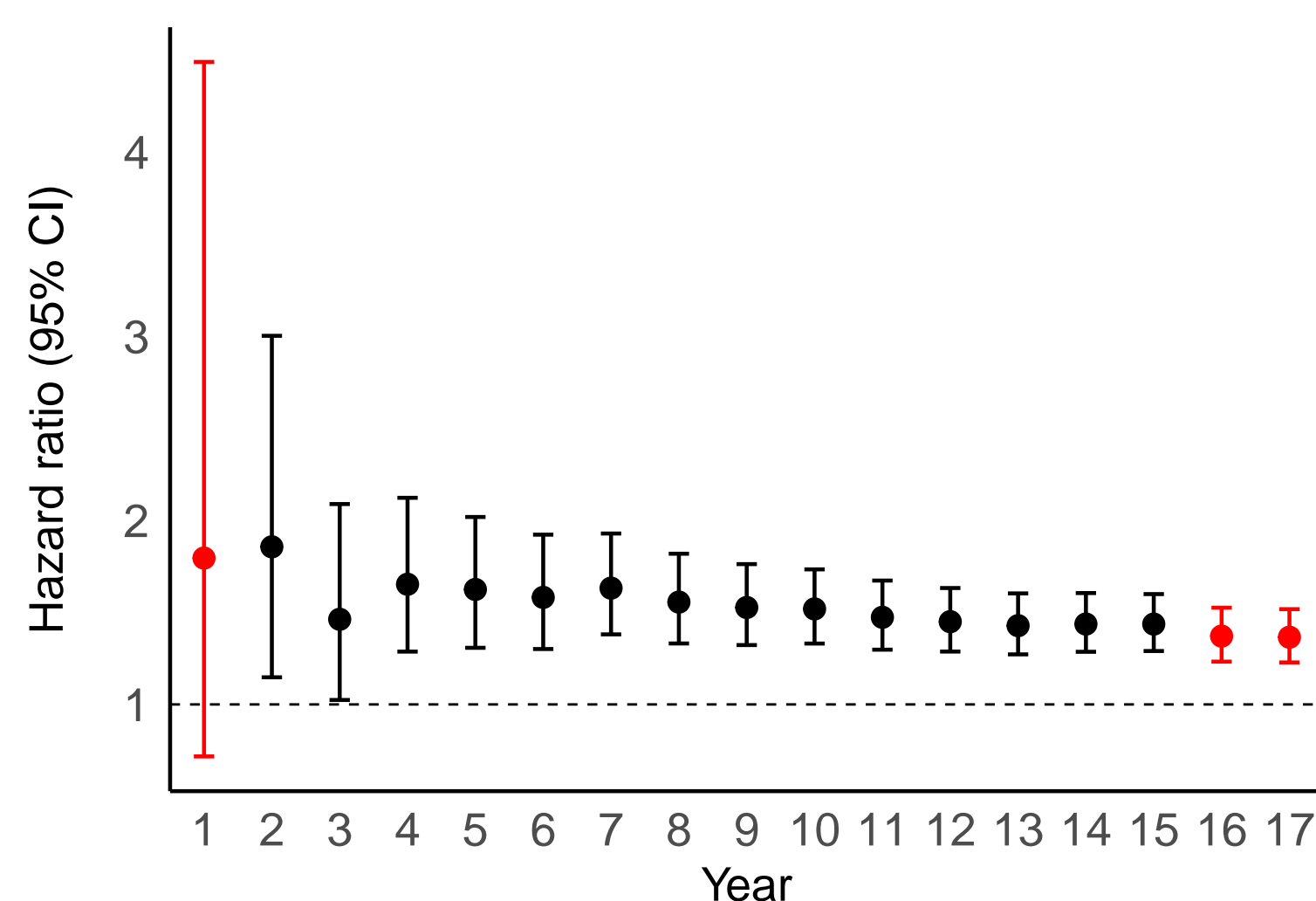

TFF3

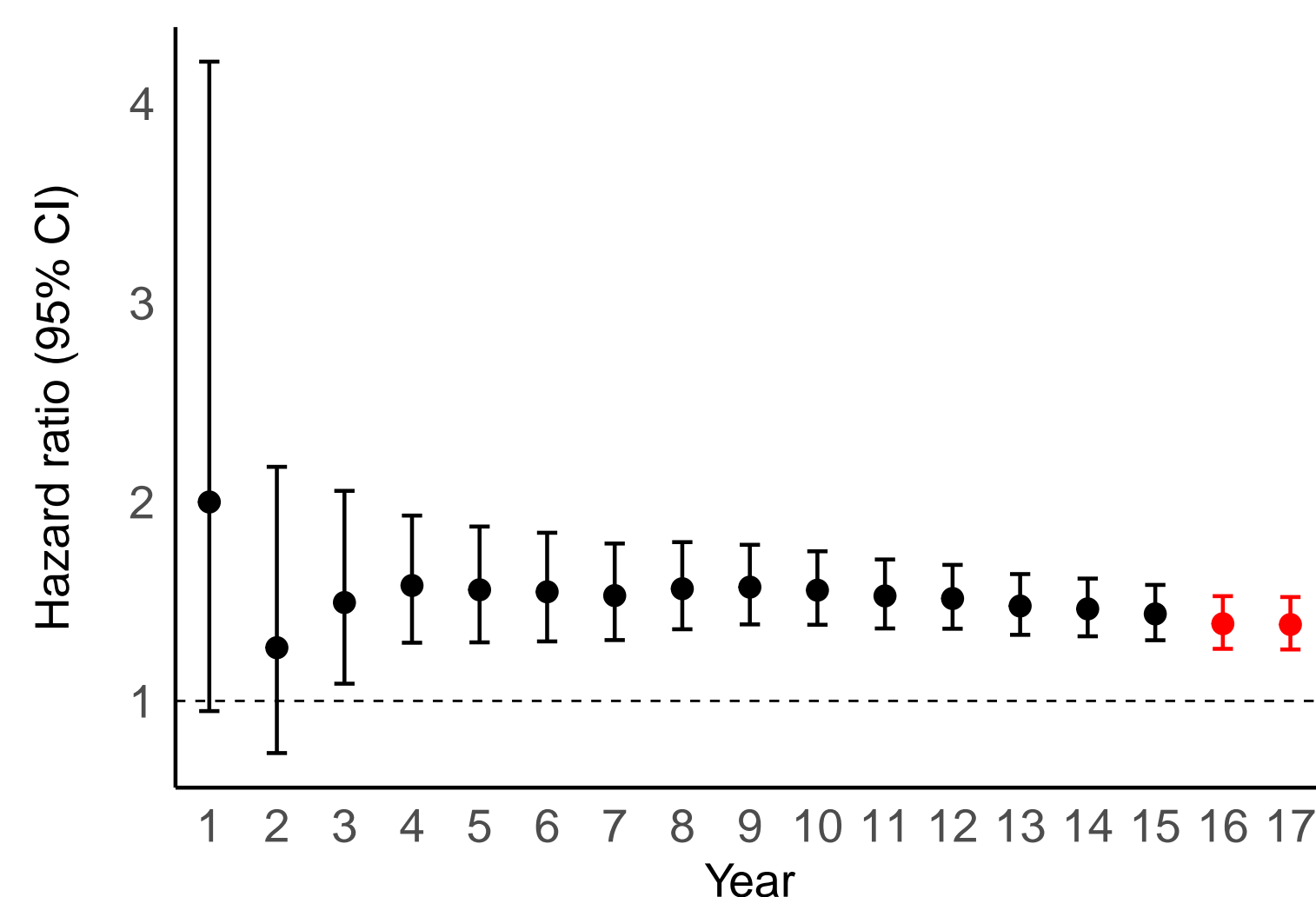

PXDN

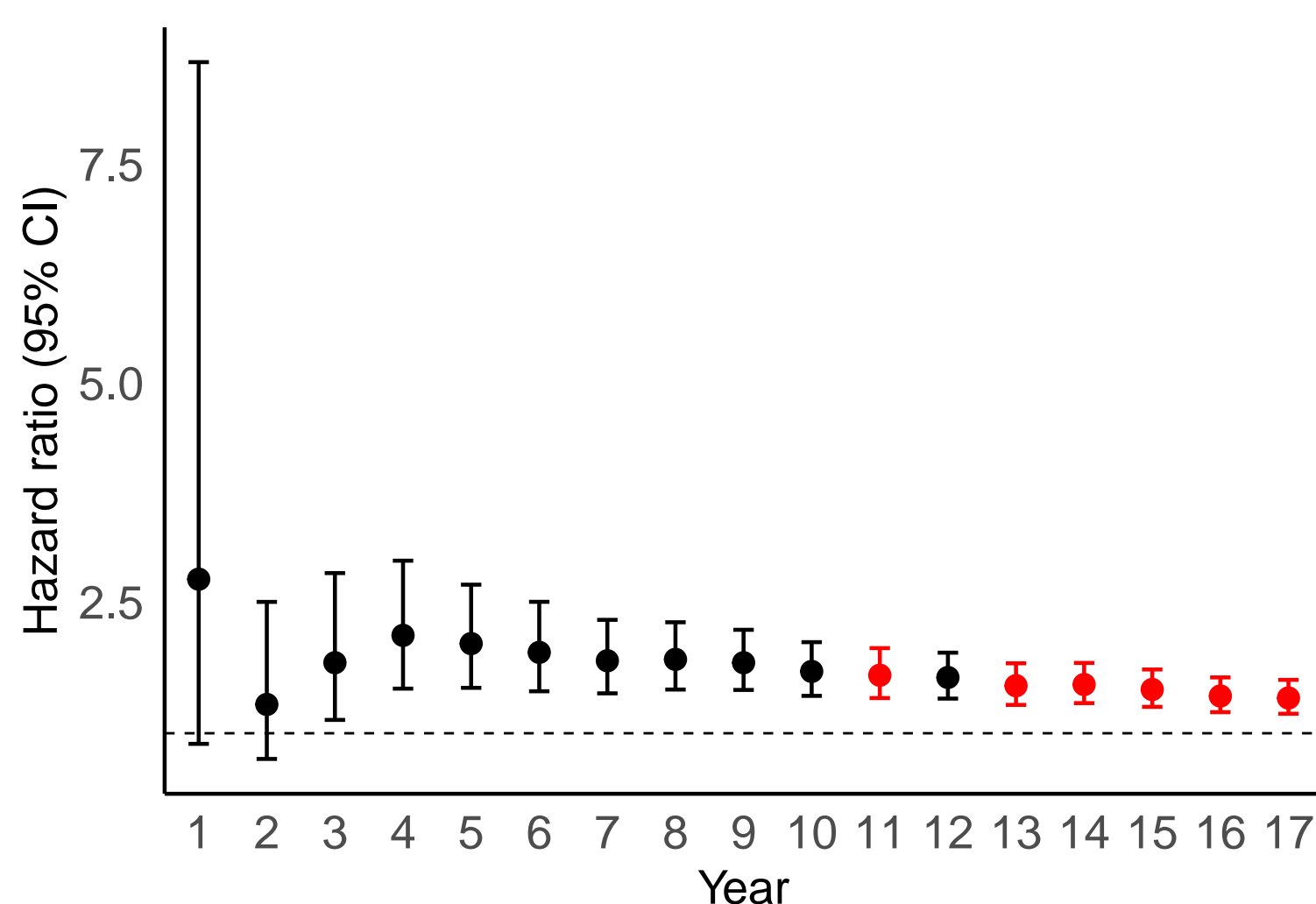

RNASE1

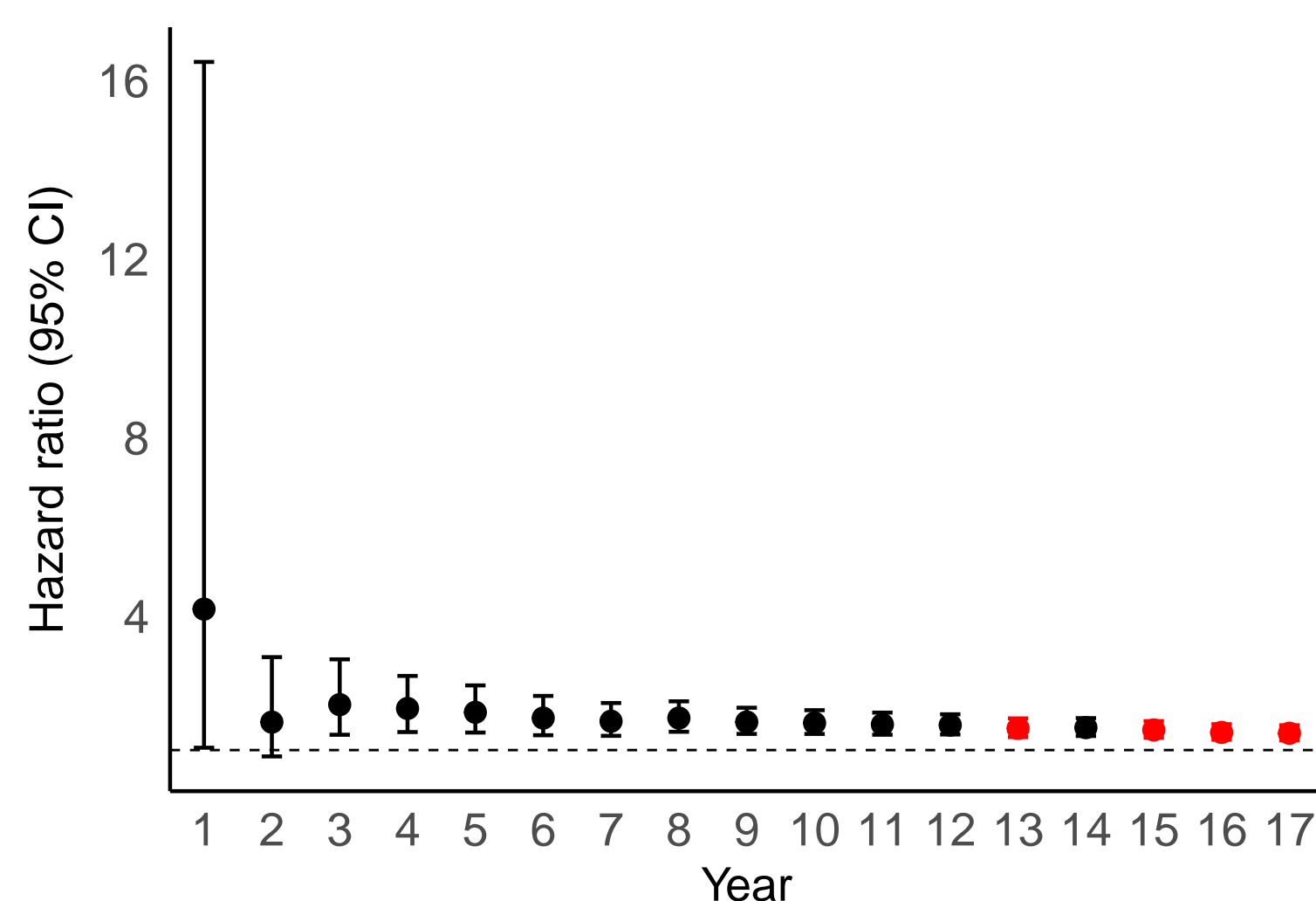

NPS

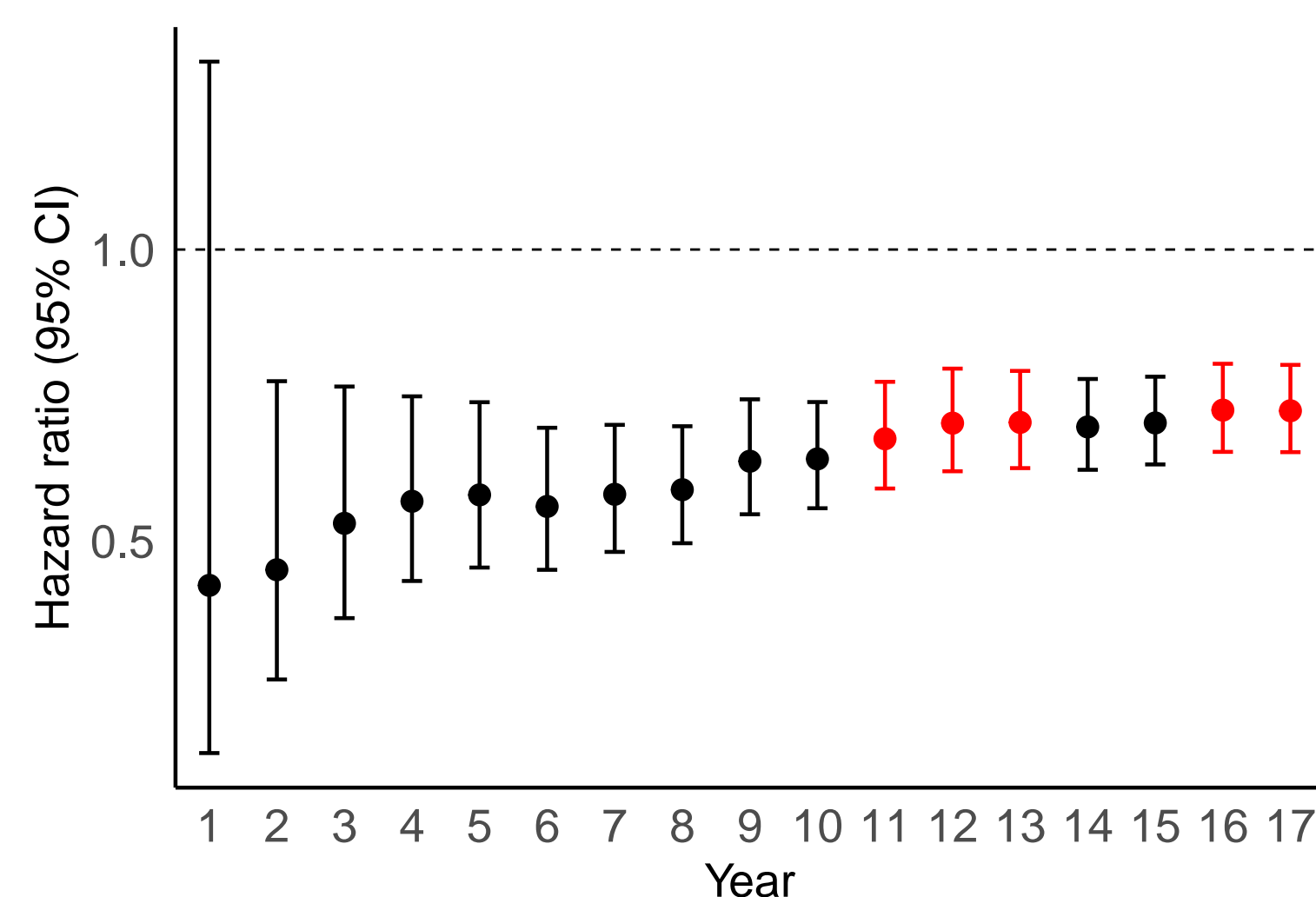

CEMP1

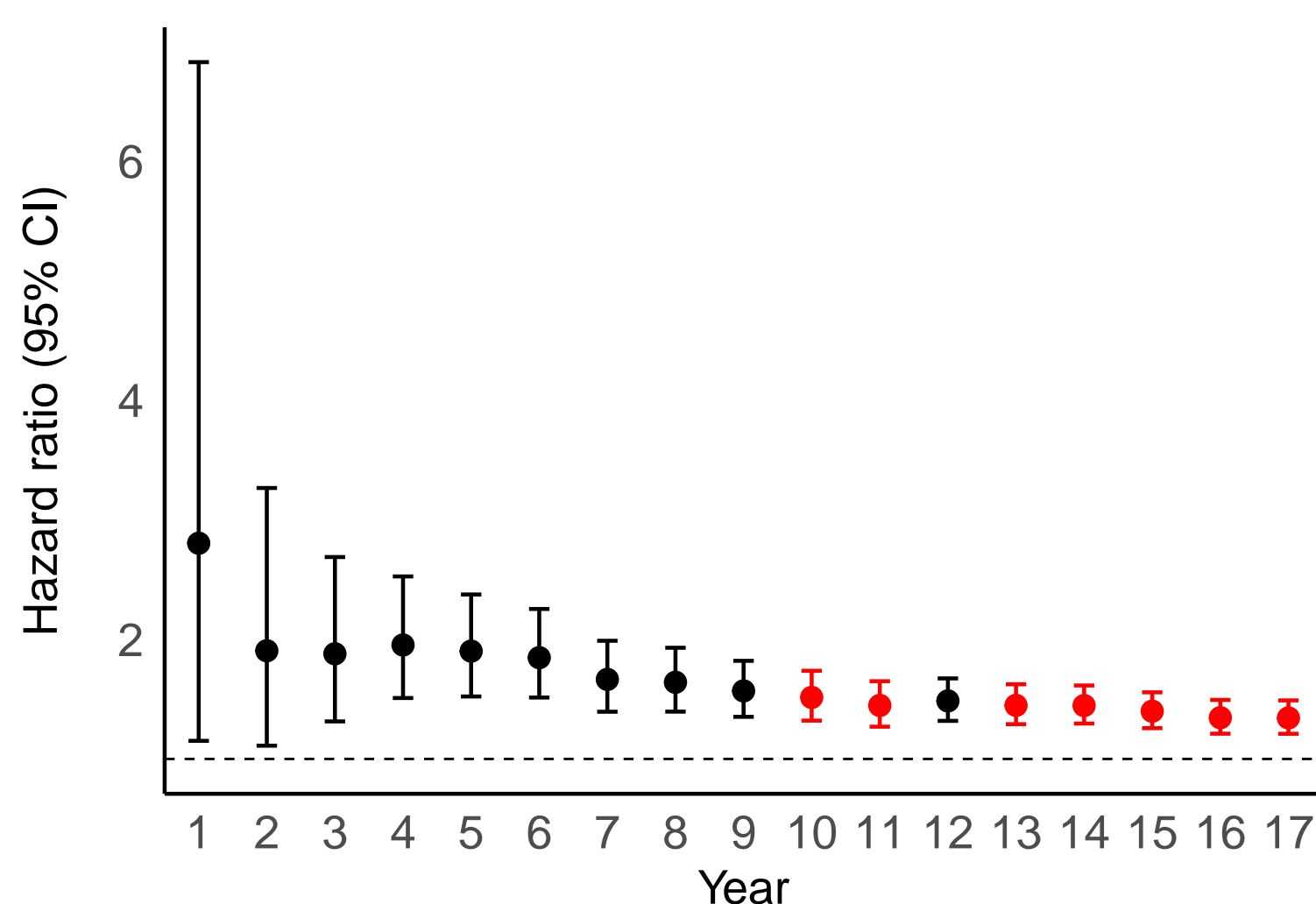

BAGE3

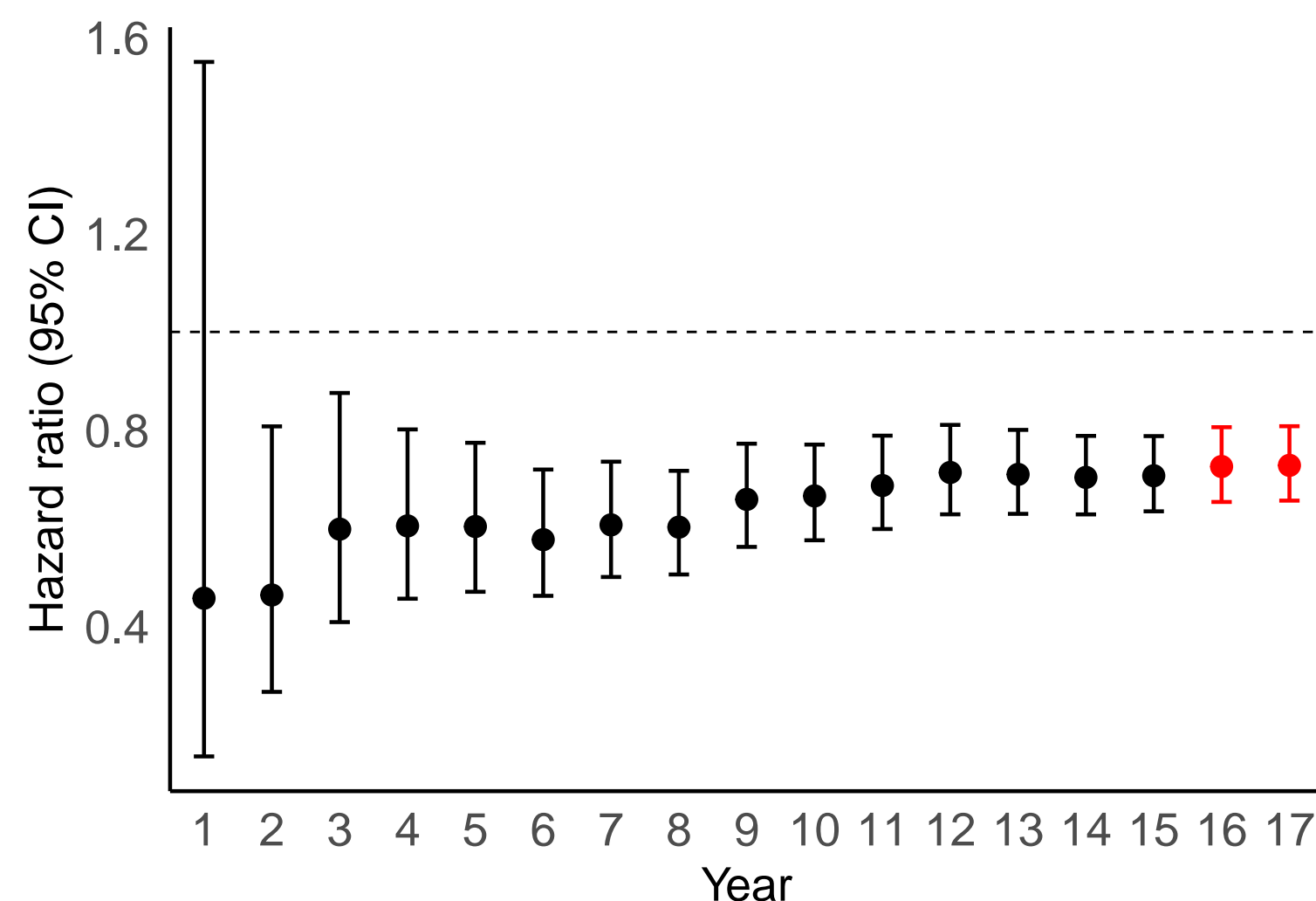

Supplement: Supplement 4 — Cox proportional hazards regression models were used to assess associations between individual SomaScan 11K plasma proteins measured at wave 2 and all-cause mortality (n = 358 deaths among 694 individuals). Models were run annually for up to 17 years of follow-up and adjusted for chronological age, sex, smoking, alcohol consumption, body mass index (BMI), and estimated glomerular filtration rate (eGFR). Forest plots show 11 of the top 20 proteins with the largest hazard ratios that violated the proportional hazards assumption (Schoenfeld residuals P < 0.05), with the year(s) of violation highlighted in red. Circles represent estimated hazard ratios, and error bars indicate 95% confidence intervals. The horizontal dotted line denotes a hazard ratio of 1. [file media-4.pdf]
