# Supplementary material for: Multimodal Ageing Biomarkers and Plasma Proteomic Signatures Associated with All-Cause Mortality
Source: medRxiv. 2026 Mar 10:2026.03.09.26347914. Preprint. [Version 1] doi: 10.64898/2026.03.09.26347914 (PMC13019151; doi:10.64898/2026.03.09.26347914)
Supplement: Supplement 3 — Cox proportional hazards regression models were used to assess associations between individual ageing biomarkers measured at wave 2 and all-cause mortality (n = 326–444 deaths in 656–861 individuals, varying by biomarker). Models were fitted annually for up to 17 years of follow-up and adjusted for chronological age and sex. Forest plots are shown only for ageing biomarkers that violated the proportional hazards assumption (Schoenfeld residuals P < 0.05), with the year(s) of violation highlighted in red. Circles represent estimated hazard ratios, with error bars indicating 95% confidence intervals. The horizontal dotted line denotes a hazard ratio of 1. [file media-3.pdf]

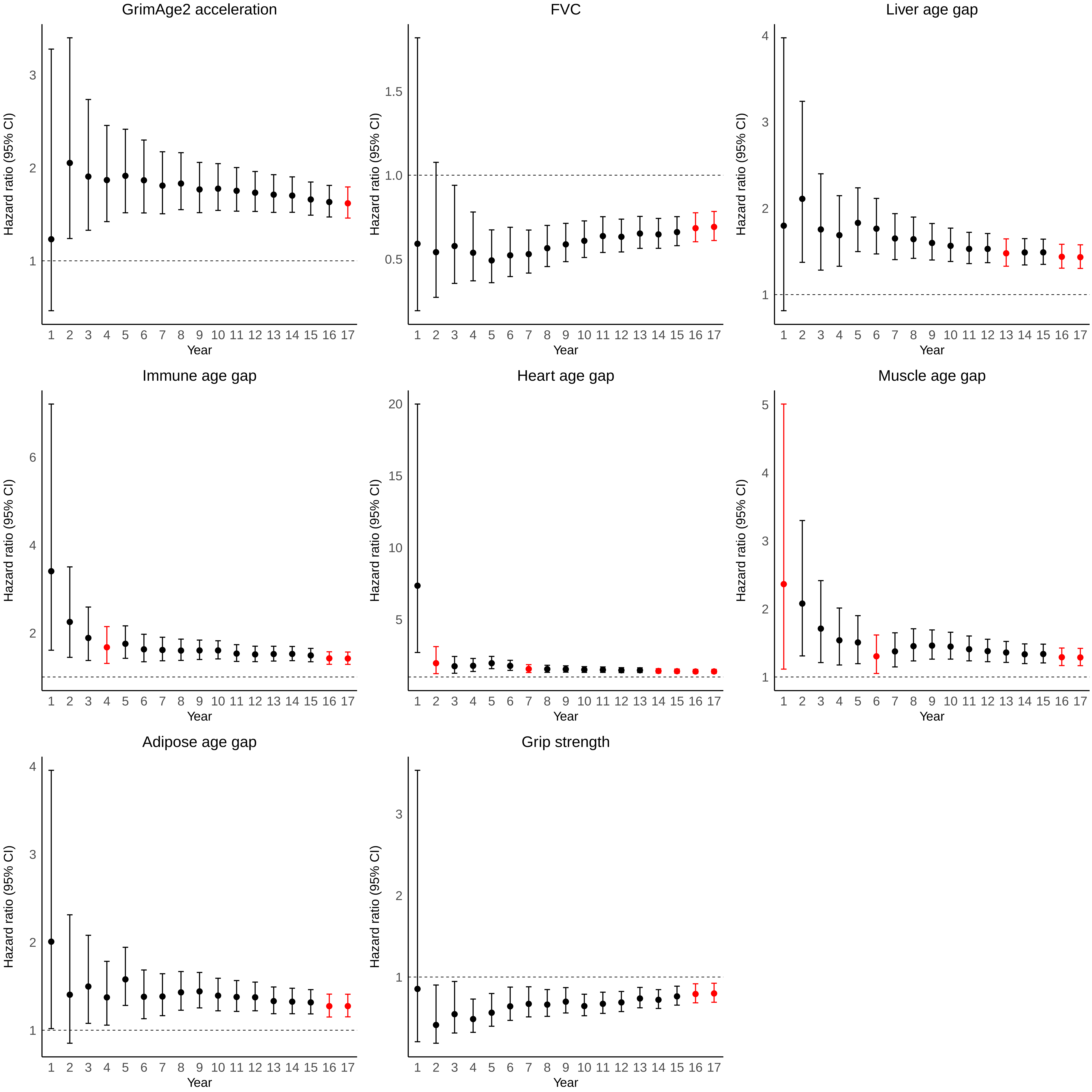
GrimAge2 acceleration
FVC
Liver age gap
Immune age gap
Heart age gap
Muscle age gap
Adipose age gap
Grip strength
Hazard ratio (95% CI)
Year
1 2 3 4 5 6 7 8 9 10 11 12 13 14 15 16 17
1 2 3
0.5 1.0 1.5
1 2 3 4
2 4 6
5 10 15 20
1 2 3 4 5
1 2 3 4
1 2 3
